# Regulatory Flexibility of Sustaining Daily Routines and Mental Health in Adaptation to Financial Strain: A Vignette Approach

**DOI:** 10.3390/ijerph18063103

**Published:** 2021-03-17

**Authors:** Wai Kai Hou, Li Liang, Clint Hougen, George A. Bonanno

**Affiliations:** 1 Department of Psychology, The Education University of Hong Kong, Hong Kong, China; 2 Centre for Psychosocial Health, The Education University of Hong Kong, Hong Kong, China; lleunglik@gmail.com; 3 Gordon F. Derner School of Psychology, Adelphi University, New York, NY 11530, USA; hougencc@gmail.com; 4 Department of Counseling and Clinical Psychology, Teachers College, Columbia University, New York, NY 10027, USA; gab38@columbia.edu

**Keywords:** financial strain, daily routines, regulatory flexibility, mental health, vignette

## Abstract

A vignette approach was adopted to investigate flexibility of sustaining daily routines and whether and how this ability was related to mental health at different levels of financial strain. Three separate studies were conducted with community-dwelling adults (*N* = 1685) in the USA. In Study 1, we drafted, tested, and modified vignettes with reference to pilot data on the relevance of the scenarios and response options. In Study 2, regulatory flexibility of sustaining daily routines, as calculated in term of context sensitivity and responsiveness to feedback, was formulated correlations with self-reported instruments to demonstrate its concurrent validity, discriminant validity, and criterion-related validity. In Study 3, path analysis examined the associations of regulatory flexibility of sustaining daily routines with psychological distress and well-being, and the moderating effects of subjective financial strain on the associations. Results showed that the inverse associations of context sensitivity and responsiveness to feedback with depressive symptoms were stronger at medium/high levels relative to lower levels of perceived financial strain. The inverse association between context sensitivity and positive affect was significant only at higher levels of strain. Our findings could provide a feasible direction for developing scalable behavioral interventions for potential mental health problems, especially among those with a lower socioeconomic status.

## 1. Introduction

Despite the fact that the USA is one of the most affluent countries in the world, there are consistent unequal distributions of income and wealth, with the richest 5% of the population owning two-thirds of the wealth [1]. At least 57.4% of families had savings below US $20,000 during 2015–2016 [2]. More specifically, among low-income adults (income ≤ 200% of the poverty level), 85.9% have US $5000 or less in family savings [3].

A growing body of evidence suggests a strong socioeconomic gradient in mental health. In an 8-wave prospective study in American working adults between 1999 and 2013, a decline in income was associated with higher levels of psychological distress [4]. Controlling for age, sex, marital status, and education level, those with at least 12 months of severe nonpsychotic mood disorders reported 55% less income relative to those without disorders [5]. Perceived financial strain, denoting individuals’ evaluation of the insufficiency of income for basic living, has been associated consistently with poorer mental health [6,7] regardless of differences in actual household income or education level [8,9].

A series of studies that were conducted in the USA or other more developed parts of the world have found a close association between financial strain and daily living. In many families, family time involves functional tasks such as cooking, hygiene, and childcare [10,11]. But in low-income families, family time is more likely to be filled with distracted and unresponsive interactions and a greater number of efforts to resolve stressors, such as negotiating the household budget [12,13]. In the face of financial strain, people are also less likely to spend time with spouses on leisure activities; reduced spousal leisure activities were found to be associated with lower relationship quality [14,15,16]. In populations affected by conflicts, disruption of regular routines of eating, sleep, socializing and leisure activities, when combined with lower socioeconomic status (i.e., education and income level), were associated with higher depressive symptoms and suicidal ideation [17].

In the current investigation, we aimed to examine the ability of sustaining daily routines from a regulatory flexibility perspective. In the sections that follow, first, the current conceptual and empirical literature on regulatory flexibility will be discussed. Then, we will introduce a new conceptualization on how to study daily routines in adaptation to different forms of stressors, followed by an application of the individual components in the regulatory flexibility perspective to explain the role of daily routines in adaptation.

### 1.1. Daily Routines: Type and Sustainment

Everyday life contains a multitude of routines and interactions with different people in our social network. A recent model suggests that sustainment of daily routines is one of the basic processes for demonstrating psychological resilience over time [18,19]. During ongoing stress people are challenged to sustain their daily routines while they are increasingly drawn to focus on the stressors or their own distress. Daily routines will either be disrupted or terminated because traumatic and chronic stress is usually associated with contexts and an ecology that restricts individuals from practicing regular daily routines. Daily routines have been parsed into two types and studied with reference to different degrees of necessity [20,21,22]. Primary daily routines refer to behaviors that are necessary for maintaining livelihood and biological needs, such as hygiene, sleep, eating, and home maintenance [23,24], whereas secondary daily routines refers to optional behaviors that are dependent upon motivation and preferences, such as exercising, leisure, social activities, and employment or work involvement [25,26].

Upholding functional daily routines can be a natural, adaptive coping process in chronic financial strain despite restrictive contexts. Low-income families often strive to create a more resourceful environment for adaptation. Conversations and problem solving with regard to financial strain, chores, childcare, and spousal conflicts have been observed across studies [27,28,29]. Three behavioral pathways could help sustain routines during stress, namely consolidation, replacement, and addition [21]. People consolidate existing routines to regain their regularity prior to a stressful encounter. While people consolidate some routines, they need to replace others that have been terminated with similar alternatives. In addition, it is also feasible to add new routines to complete the everyday life structure. Consolidation and addition can be a proactive, reactive, or continuous procedure, throughout adaptation and before and after termination of routines. Replacement is reactive to terminated routines when consolidation fails to work. Disruption and termination of routines can also co-occur. 

### 1.2. Regulatory Flexibility

Effectiveness and efficacy of sustaining daily routines could be evaluated in terms of different components of regulatory flexibility [30]. Flexible coping and emotion regulation unfold over time in sequential components. Two of these components, sensitivity to context and responsiveness to feedback, suggest applications to the task of sustaining daily routines. Sensitivity to context refers to the degree to which individuals can accurately perceive and understand situational demands and opportunities. The most effective regulatory strategies are the ones that match with situational demands. Responsiveness to feedback refers to the degree to which individuals are able to evaluate their chosen strategies in accordance with situational demands, discontinue ineffective strategies, and re-engage in alternative, effective strategies. 

These components tend to be inter-related and have shown predictive relationships to adjustment and psychopathology. For example, a greater ability to detect the presence of cues for regulation correlates with a greater flexibility in coping and emotion regulation, whereas a higher ability to detect an absence of cues correlates with lower anxiety and depressive symptoms [31]. Apart from its direct association with psychopathology, context sensitivity can also compensate for deficits in the flexible regulation of facial expressions of emotion [32]. Among school-aged children, sensitivity to contextual cues for regulating anger and fear was generally observed, whereas sensitivity to contextual cues for regulating sadness tended to emerge in later childhood [33]. People with chronic pain who were less sensitive to contextual cues for interpersonal interactions also reported more problems relating to their pain such as higher intensity, frequency, and disability and less effective coping behaviors [34]. The experience of intense negative emotion has been found to provide feedback for switching emotion regulation strategies [35]. Among those with higher scores on a combined measure of responsiveness to internal feedback (e.g., self-report, heart rate, skin conductance), frequent strategy switching predicted a higher well-being. 

### 1.3. The Present Study

In the current investigation, we used a vignette approach to adapt the context sensitivity and responsiveness to feedback components to assess individuals’ ability to adjust to changes in daily living due to financial difficulties. A vignette is a brief description of a person or situation written with the aim of simulating specific realistic features of real-world settings [36]. Based on the sociodemographic characteristics and lifestyles of the U.S. citizens, we opted for a vignette approach because traditional self-report instruments of perceived ability are limited by possible response biases and deficits in self-knowledge on a specified domain [37,38]. Assessing merely feelings and perceptions of ability also limit the application of research findings to only those interventions that focus on change in concepts and perceptions [39,40]. By contrast, the hypothetical scenarios used in a vignette approach are less susceptible to response biases, do not require accurate self-evaluations of one’s own ability, and allow for application to a broader range of interventions.

In the present study (Table S1 in Supplementary Materials), vignettes were initially drafted, tested, and modified with reference to the pilot data on the relevance of the scenarios and response options among a community sample (Study 1). Second, the validity of regulatory flexibility of sustaining daily routines, calculated in terms of context sensitivity and responsiveness to feedback, were examined (Study 2). Third, path analysis was used to test the associations of individual differences in the ability of context sensitivity and responsiveness to feedback of sustaining daily routines for financial strain with psychological distress and well-being, and the moderating effects of subjective financial strain on the associations (Study 3). To reduce the assessment load on the participants, different instruments were administered to four separate samples. Table 1 summarizes the characteristics of the four samples.

## 2. Study 1: Development of the Vignette Task

Under immediate deprived conditions, such as financial difficulty due to job loss, mass conflicts, and natural disasters, disruption and termination of primary routines could lead directly to rapid breakdown of the overall regularized daily living, whereas disruption and termination of secondary routines could only reduce overall resourcefulness, leading to gradual breakdown of daily living [21,22,41,42]. Therefore, sustainment of primary routines should be prioritized over secondary routines. In addition, resources are needed for consolidation, replacement, and addition [20,43,44]. But the amount of resources invested in each routine is disproportional. Fewer resources are needed for consolidating disrupted routines compared with the replacement of terminated routines, because termination of routines requires the building up of alternative routines from scratch. There is evidence showing that under higher perceived stress, consolidation of existing social ties was preferred over the addition of new communication ties [45]. Striving for safety and preservation to ensure non-losses prevails under adverse stressors that impair performance, whereas striving for growth and accomplishment to ensure gains is preferred under a benign challenge that offers opportunities [46,47]. 

With the adaptive priority of (1) primary over secondary routines and (2) disruption over termination, the goal of Study 1 was to develop vignettes that simulate realistic changes in daily routines in the face of financial strain and realistic responses. The context sensitivity and responsiveness to feedback components of regulatory flexibility guided the development of each vignette, which encompassed three components, namely scenario, changes, and standardized responses. The scenario described the financial strain experienced by an individual. Two changes in daily routines were also included. Responses with respect to the changes were written. 

### 2.1. Scenario

A pool of 24 scenarios were drafted to reflect financial strain, with 4 scenarios randomly assigned on each of the following combinations of routines changes: (1) primary-disrupted, primary-terminated; (2) primary-disrupted, secondary-disrupted; (3) primary-disrupted, secondary-terminated; (4) primary-terminated, secondary-disrupted; (5) primary-terminated, secondary-terminated; (6) secondary-terminated, secondary-disrupted. The actor in each scenario was described with a name, gender, age, full-time/part-time job, relationship, and with/without children. Based on a review of previous studies using a vignette task [48], all scenarios were drafted and edited based on the following criteria: (1) a maximum of 3 sentences for each scenario; (2) the absence of explicit emotionally charged words; (3) financial strain as the focal stressor; and (4) a job randomly drawn from a list of low-income jobs (e.g., barista, retail salesperson, housekeeper, janitor, etc.) We also randomly assigned age (18–70 years), gender (female/male), job nature (full-time/part-time), and family status (having children or not) to all 24 vignettes. Sample scenario: 

“Levy is a 46 years old employee of a car wash company. Levy’s partner is also employed. Though they have joint income, they struggle to make ends meet each month, and so, Levy is constantly on the search for more work hours or a second job.” 

### 2.2. Routines Changes

#### 2.2.1. Measure

As each of the 24 scenarios contained 2 daily routines changes, a pool of 48 daily routines changes were generated. Sample daily routines changes: “You used to sleep well at night and take naps during the day. Now, you take naps or gets good sleep at night only occasionally.” (Disrupted primary routines); “You used to do a lot with friends each month. Now, you cut back on the amount of time spent with friends.” (Disrupted secondary routines); “You used to regularly sleep well. Now, you often go sleepless.” (Terminated primary routines); and “You used to hang out with friends at the local bar/café/restaurant on a regular basis. Now, you can no longer meet friends there.” (Terminated secondary routines). 

#### 2.2.2. Participants, Procedure, and Results

After obtaining ethics approval from the Institutional Review Board for the Protection of Human Subjects at Teachers College, Columbia University (IRB number: 17-351), the survey session was advertised on Amazon’s Mechanical Turk (MTurk) service (MTurk) as “A survey about daily stress” and consisted of demographic items and the draft items for realistic changes of daily routines. MTurk facilitates experimental and survey data collection using crowdsourced convenience samples. There is evidence showing that data derived from MTurk participants is valid, representative [49], and reliable [50,51]. 

The accuracy of the routines changes on describing the reality were examined by a sample of 356 people (166 females) aged between 18 and 73 years (*M* = 34.5, *SD* = 10.9). To minimize multiple participations, we restricted each MTurk Worker ID to one participation only. Participants were paid US $3.10 for their participation. In the survey, the instructions read, “Below are several real-life changes some people go through. Rate to what extent these changes accurately describe the reality.” Participants rated each item on a 5-point Likert scale ranging from 0 (Not at all) to 4 (Very much). Ratings of the items were averaged. The 48 changes ranged from 2.35 to 2.84 with median score of 2.52 and mean score of 2.53 (*SD* = 0.11).

### 2.3. Response

Following presentation of a scenario with two daily routines changes, participants would rank five standardized responses in reaction to the changes. Two responses were consolidation and/or replacement of the two daily routines changes. Two responses were consolidation and/or replacement and addition of routines that were not the two changes. The fifth response option indicated no action. For example, with disrupted hygiene and terminated social activity, the responses would be as follows:

1. I would work on showering more regularly. (Consolidate/replace disrupted primary routines)
2. I would start meeting friends again. (Consolidate/replace terminated secondary routines)
3. I would get more regular exercise. (Consolidate/replace unrelated routines)
4. I would start spending more time on relaxation. (Add unrelated routines)
5. I would not do anything.

In this item, according to the predicted adaptive priority of primary routines and disruption/termination, consolidation of personal hygiene (i.e., disrupted primary) is prioritized over replacement of social activity (terminated secondary), followed by consolidation of unrelated routines (i.e., exercise) and then addition of unrelated routines (i.e., leisure). The ranking indicated context sensitivity in each item.

Monitoring feedback is about the efficacy of the regulatory strategy that has been enacted. Based on the feedback, individuals prioritize, again, consolidation, replacement, and addition of routines if necessary [30]. If the participant ranked correctly, she/he would be directed to sub-question 1 “If after doing the above things you find that you feel better physically and mentally, how would you prioritize them now?” and rank the behaviors again. If the participant ranked incorrectly, then she/he would be directed to sub-question 2 “If after doing the above things you find that you do not feel better physically and mentally, how would you prioritize them now?” Rankings on the two sub-questions indicated responsiveness to feedback. The sample of a complete vignette is presented in Appendix A.

## 3. Study 2: Examining Validity of Regulatory Flexibility of Sustaining Daily Routines

Following development of the vignette task for assessing context sensitivity and responsiveness to feedback in sustaining daily routines in time of financial strain, Study 2 aimed to investigate the concurrent validity, discriminant validity, and criterion-related validity of the vignette task.

### 3.1. Methods

#### 3.1.1. Participants and Procedure

Data were extracted from different measurements in two study samples (sample 2 and 3). A total of 791 participants (sample 2: 95 females; sample 3: 187 females) aged between 18 and 72 years completed the measures and were paid US $3.20 for their participation. Each MTurk Worker IDs was restricted to one participation and thus did not duplicate across the two surveys. The study protocol was approved by the Institutional Review Board for the Protection of Human Subjects at Teachers College, Columbia University (IRB number: 17-351).

#### 3.1.2. Measures

##### Regulatory Flexibility in Sustaining Daily Routines

One item was randomly selected from the four scenarios in each of the six combinations. A total of 6 vignettes were used to assess context sensitivity and responsiveness to feedback, each of which was scored using rankings of actions towards daily routines changes during financial strain. In each item, the first ranking indicated context sensitivity, whereas the second ranking indicated responsiveness to feedback. The standard rankings for the vignettes were formulated based on the aforementioned adaptive priority. Spearman’s rank correlation coefficient assesses how well participants’ rankings matched with the standard rankings, ranging from −1 (perfect inverse correlation) to 1 (perfect positive correlation) with 0 indicating no correlation. Context sensitivity was indicated by averaging correlation coefficients in all the first rankings whereas responsiveness to feedback was indicated by averaging correlation coefficients in all second rankings. Higher Spearman’s rank correlation coefficients indicated higher levels of context sensitivity or responsiveness to feedback.

##### General Ability of Context Sensitivity

The 20-item Context Sensitivity Index (CSI) assessed the general ability to perceive cues of contextual demands [31]. It includes two separate indices to capture sensitivity to the presence of contextual cues (Cue Presence index) and to the relative absence of cues (Cue Absence index). Participants answered 20 scenario-based questions on a 7-point scale (1 = Not at all, 7 = Very much). Cue Presence index was calculated by summing 10 items. Cue Absence index was calculated by summing another 10 items after being reversed. CSI assesses perception of different contextual cues across diverse scenarios and thus each item represents a unique aspect of the latent construct, whereas Cronbach’s alpha reflects internal consistency for items that represent a version of the same underlying construct [31]. Alpha was not calculated for CSI.

##### Social Problem-Solving Ability

The Social Problem-Solving Inventory-Revised Short-Form (SPSI-R: SF) was used to assess social problem-solving ability [52,53]. SPSI-R: SF measured five dimensions of social problem-solving ability, namely positive problem orientation (PPO), negative problem orientation (NPO), rational problem solving (RPS), impulsiveness/carelessness style (ICS), and avoidance style (AS), each of which comprised five items. Participants reported to what extent each item was true of their thoughts, feelings, or behaviors when solving problems in real life on a 5-point scale from 1 (not at all true of me) to 5 (extremely true of me). Items of NPO, ICS, and AS were reversely coded before a sum score was calculated to represent general problem-solving ability. Cronbach’s alpha was 0.90.

##### Regularity of Daily Routines

Sustainability of Living Inventory (SOLI) was utilized to measure regularity of daily routines [22]. SOLI contained 42 items assessing eight dimensions of daily routines: hygiene, eating, sleep, duties at home, leisure at home, exercising, social activities, and work/study involvement. Participants rated how regularly they do the 42 activities every day on a 3-point scale from 0 (not at all regular) to 2 (very much regular). A score indicating regularity of daily routines was calculated by averaging the scores on 42 items. In the current administration, Cronbach’s alpha was 0.92.

##### Flexible Emotion Regulation

The 16-item Flexible Regulation of Emotional Expression (FREE) Scale assessed the ability to flexibly regulate emotional expression [54]. The scale consisted of four subscales measuring the abilities to enhance and suppress expressions of positive emotions and negative emotions. Participants rated how well they could either “be even more expressive than usual of how you were feeling” or “conceal how you were feeling” in a given scenario (e.g., “Your friend is telling you about what a terrible day they had.”) on a 6-point scale (1 = Unable, 6 = Very able). A summed score was calculated across the 16 items. A higher score indicates greater flexibility in regulating emotional expressions. Cronbach’s alpha was 0.86 in the current administration.

##### Posttraumatic Stress Disorder Checklist-Civilian Version (PCL-C)

The PCL-C contains 17 items addressing the three DSM-IV diagnostic criteria for PTSD [55], which are re-experiencing, avoidance, and hyperarousal. Each symptom was evaluated with a 5-point scale from 1 (“Not at all”) to 5 (“Extremely”). A total score was calculated to represent overall severity of PTSD symptoms. Higher scores indicated greater posttraumatic symptomatology. In the current administration, the Cronbach’s alpha was 0.97.

#### 3.1.3. Analytic Plan

Concurrent validity was established by quantifying the correlations of regulatory flexibility in sustaining daily routines with CSI [31] and SPSI-R: SF [52]. We expected that moderate to strong correlations would be found among them as context sensitivity and responsiveness to feedback in sustaining daily routines indicated an ability to perceive cues to contextual demands (CSI) and ability of handling difficulties and challenges in daily life (SPSI-R: SF). Discriminant validity was demonstrated by estimating the correlations of regulatory flexibility in sustaining daily routines with SOLI [22] and FREE [54]. We anticipated that weak or null correlations would be observed because SOLI reflected one’s regular behavioral patterns and FREE assessed regulation of emotion expression, both of which may only be weakly related to the cognitive ability in sustaining daily routines, as assessed in the current vignette task. Finally, criterion-related validity was tested by deriving the correlations of regulatory flexibility in sustaining daily routines with PCL-C [55]. It was expected that the regulatory flexibility in sustaining daily routines was inversely correlated with PTSD symptoms.

### 3.2. Results

The results on concurrent, discriminant, and criterion-related validity are summarized in Table 2. As expected, concurrent validity was demonstrated by the significant, moderate positive correlations of context sensitivity and responsiveness to feedback with a CSI cue absence index (0.357 to 0.497), a CSI cue presence index (0.341 to 0.389), as well as a social problem-solving inventory (0.403 to 0.497). Discriminant validity of regulatory flexibility in sustaining daily routines was shown on the weak correlations with SOLI (0.127 to 0.152) as well as non-significant correlations with FREE (0.008 to 0.050). Criterion-related validity was demonstrated by the significant, moderate inverse correlations of context sensitivity and responsiveness to feedback with PCL-C (−0.389 to −0.452). To take into account the potential effect of gender on the study variables, we re-analyzed the associations between regulatory flexibility in daily routines and other variables with gender included as one of the covariates. The directions and magnitudes of the associations were consistent with those in the original analysis without gender as a covariate (Table S2 in Supplementary Materials).

## 4. Study 3: Sustainment of Daily Routines and Psychological Distress and Well-Being

Study 3 examined the association between sustainment of routines and mental health and the moderating effect of subjective financial strain in the association. Subjective financial strain, denoting individuals’ evaluation of the insufficiency of income for basic living, has been consistently associated with mental health across different populations [6,7,8,9,56]. In this study, path analysis was used to test the associations of individual differences in context sensitivity and responsiveness to feedback in sustaining daily routines with psychological distress (i.e., anxiety and depressive symptoms) and well-being (i.e., positive affect and life satisfaction). The moderating effects of perceived financial strain in the associations of context sensitivity and responsiveness to feedback with psychological distress and well-being were analyzed. We expected that context sensitivity and responsiveness to feedback would be inversely associated with psychological distress and positively associated with psychological well-being; perceived financial strain will be positively associated with psychological distress and inversely associated with psychological well-being. The associations of context sensitivity and responsiveness to feedback with psychological distress and well-being will be stronger at higher levels of perceived financial strain compared with lower levels of perceived financial strain.

### 4.1. Methods

#### 4.1.1. Participants and Procedure

With ethics approval from the Institutional Review Board for the Protection of Human Subjects at Teachers College, Columbia University (IRB number: 17-351), data collection was conducted using the MTurk Service. Sample 4 was used in the current study. A total of 538 participants (258 females) aged between 18 and 79 years (*M* = 35.2, *SD* = 10.9) completed the measures and were paid US $3.20 for their participation. The MTurk Worker IDs was restricted to one participation and did not duplicate those in Study 1 and Study 2.

#### 4.1.2. Measures

##### Regulatory Flexibility in Sustaining Daily Routines

Scores for each item were calculated based on the scoring method mentioned in Study 2. Context sensitivity was indicated by averaging correlation coefficients in all first rankings whereas responsiveness to feedback was indicated by averaging correlations coefficients in all second rankings. 

##### Perceived Financial Strain

A 5-item measure developed by Pearlin and Lieberman [57] was used to measure perceived financial strain. Participants rated how often they did not have enough money to afford food, medical care, clothing, and family leisure activities on a 4-point scale (1 = Almost never/never, 2 = Sometimes, 3 = Often, 4 = Almost always). A fifth item assessed the participants’ financial status at the end of the month on a 3-point scale (1 = Some money left over, 2 = Just enough money to make ends meet, 3 = Not enough money to make ends meet). Item scores were standardized and then averaged. Cronbach’s alpha was 0.77 in the current administration.

##### Anxiety Symptoms

The 6-item state version of the State-Trait Anxiety Inventory (STAI-6) was used to assess anxiety symptoms [58]. Participants rated the extent to which they felt calm, tense, upset, relaxed, content, and worried at the time of survey on a 4-point scale (1 = Not at all, 2 = Somewhat, 3 = Moderately, 4 = Very much). Scores on three positive-worded items were reverse-coded before the summed scores were calculated (range = 6–24). In this study, Cronbach’s alpha was 0.80. 

##### Depressive Symptoms

The 9-item Patient Health Questionnaire (PHQ-9) [59] was used to assess depressive symptoms over the past two weeks on a 4-point scale (0 = Not at all, 1 = On several days, 2 = More than half the days, 3 = Nearly every day). Summed scores were calculated. Higher scores indicated a higher level of depressive symptoms (range = 0–27). Cronbach’s alpha was 0.94 in the current administration.

##### Positive Affect

Positive affect was measured by the positive affect subscale of Positive and Negative Affect Scale (PANAS) [60]. Participants rated to what extent they felt ten positive emotions over the past two weeks on a 5-point scale (1 = Very slightly or not at all, 2 = A little, 3 = Moderately, 4 = Quite a bit, 5 = Extremely). Total summed scores were calculated (range = 10–50). Higher scores indicated higher levels of positive affect. Cronbach’s alpha was 0.93 in the current study.

##### Life Satisfaction

Satisfaction with Life Scale (SWLS) assessed participants’ well-being in terms of satisfaction and fulfillment of their current state [61]. Participants indicated agreement with total 5-item on a 4-point scale (1 = Strongly disagree, 4 = Strongly agree). Scores were calculated by summing across the items (range = 5–20). Cronbach’s alpha was 0.90 in the current administration.

#### 4.1.3. Analytic Plan

Zero order correlation and Mann–Whitney U tests were utilized to identify potential confounding demographic variables. Path analysis was performed using a lavaan package in R [62] to test the associations of context sensitivity, responsiveness to feedback, perceived financial strain, and their interactions with all outcome variables simultaneously. Demographic covariates and covariances between outcome variables and between predictor variables were controlled for in all models. Paths of non-significant associations between demographic covariates and outcome variables were trimmed in the final model. Summed scores of the outcome variables were used as observed variables in the models. Two interaction terms context sensitivity × perceived financial strain and responsiveness to feedback × perceived financial strain were included to test the moderation effects of perceived financial strain on the associations of context sensitivity and responsiveness to feedback with outcome variables. To avoid multicollinearity, standardized scores were used in the calculation of the interaction terms. Simple slope tests on significant interaction effect(s) were conducted to examine whether the simple slopes of the interaction term(s) were different from zero. Higher and lower levels of perceived financial strain were indicated by scores at one SD above/below the mean, whereas medium levels were within one SD of the mean. Model fit was evaluated using comparative fit index (CFI), Tucker–Lewis index (TLI), root mean square error of approximation (RMSEA), and standardized root mean square residual (SRMR). CFI and TLI values ≥ 0.90 and RMSEA and SRMR values ≤ 0.08 indicated a good fit [63].

### 4.2. Results

#### 4.2.1. Perceived Financial Strain

As shown in Table 3, the majority of participants experienced varying degrees of difficulty (sometimes/often/almost always) in affording food (60.8%), medical care (69.1%), clothing (69.1%), and family leisure activities (76.8%). About 65% of the participants either had not enough money to make ends meet or had just enough money to make ends meet. Perceived financial strain was not associated with annual household income level (*r* = −0.019, *p* = 0.664).

#### 4.2.2. Basic Model

Descriptive statistics and correlations of the study variables are summarized in Table 4. The model with context sensitivity of sustaining routines, responsiveness to feedback of sustaining routines, perceived financial strain, and the four outcomes demonstrated acceptable model fit: CFI = 0.96, TLI = 0.91, RMSEA = 0.06 (95% confidence interval (CI): 0.04, 0.08), and SRMR = 0.05. Context sensitivity was significantly inversely associated with anxiety symptoms (*β* = −0.22, 95% CI: −0.32, −0.12) and depressive symptoms (*β* = −0.35, 95% CI: −0.43, −0.26). No significant association was found between responsiveness to feedback and outcomes. Perceived financial strain was positively associated with depressive symptoms (*β* = 0.19, 95% CI: 0.12, 0.26).

#### 4.2.3. Moderation Model

Model fit remained acceptable after adding the two interaction terms: CFI = 0.96, TLI = 0.91, RMSEA = 0.06 (95% CI: 0.04, 0.07), and SRMR = 0.06. Perceived financial strain was significantly associated with depressive symptoms (*β* = 0.23, 95% CI= 0.16, 0.30) and positive affect (*β* = 0.10, 95% CI = 0.01, 0.18). Context sensitivity was significantly inversely associated with anxiety symptoms (*β* = −0.20, 95% CI: −0.31, −0.10) and depressive symptoms (*β* = −0.30, 95% CI: −0.38, −0.21). No significant association was observed between responsiveness to feedback and outcomes. Context sensitivity × perceived financial strain was significantly inversely associated with depressive symptoms (*β* = −0.09, 95% CI: −0.17, −0.01) and positive affect (*β* = −0.11, 95% CI: −0.21, −0.01). Responsiveness to feedback × perceived financial strain was significantly inversely associated with depressive symptoms (*β* = −0.09, 95% CI: −0.17, −0.01). No significant association was found between the interaction terms and anxiety symptoms and life satisfaction. Age (*β* = −0.20 to −0.13), income level (*β* = −0.15 to 0.20), education level (*β* = 0.14 to 0.16), marital status (*β* = 0.16 to 0.24), and employment status (*β* = 0.07) were included as exogenous confounding variables in both models (all *p*s ≤ 0.041).

**Figure 1:**
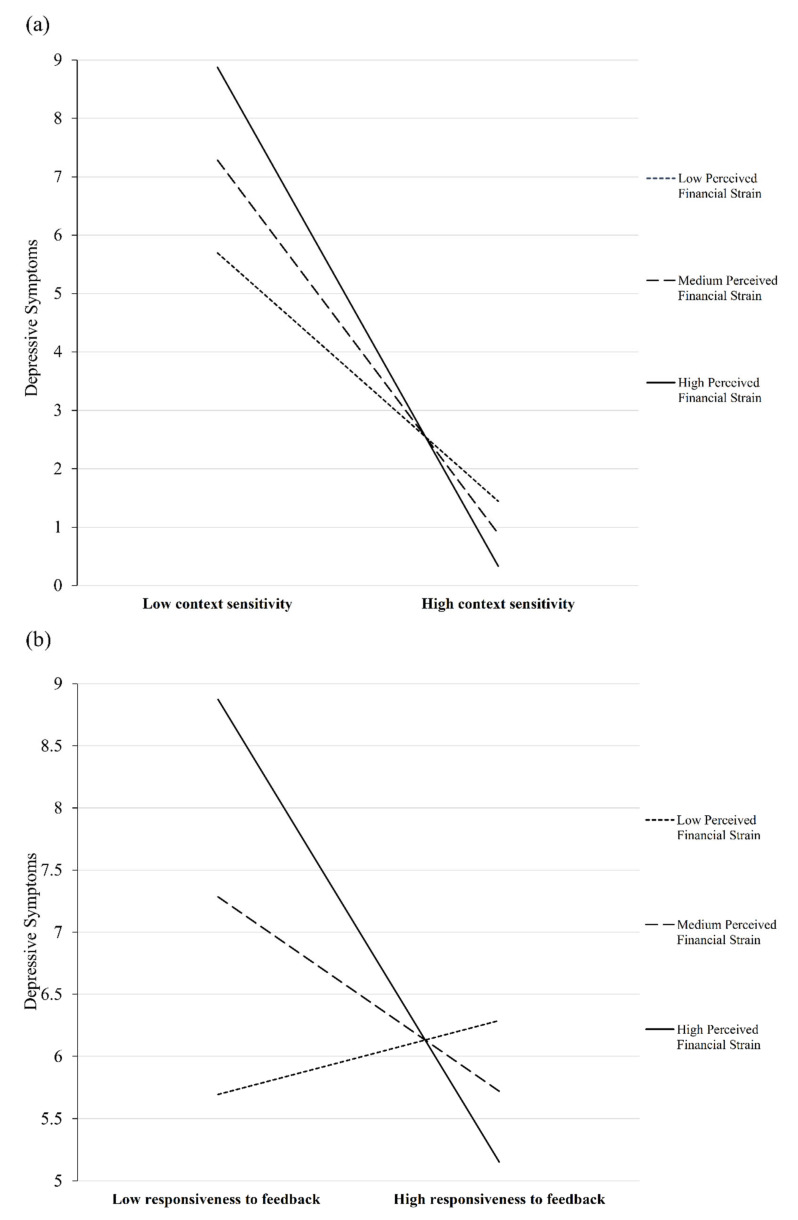
Plot of the moderating effect of perceived financial strain on the association of context sensitivity (**a**) and responsiveness to feedback (**b**) in routines with depressive symptoms.

**Figure 2:**
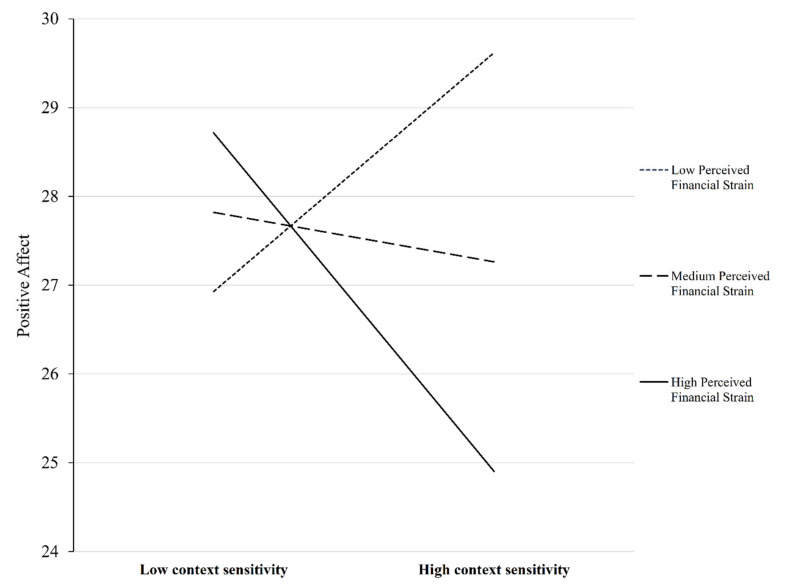
Plot of the moderating effect of perceived financial strain on the association between context sensitivity in routines and positive affect.

Simple slope tests revealed that the inverse association between context sensitivity and depressive symptoms were all significant at low (*β* = −0.21, *p* = 0.008), medium (*β* = −0.30, *p* < 0.001), and high (*β* = −0.39, *p* < 0.001) levels of financial strain, the association was stronger as the perceived financial strain increased (Figure 1a). Additionally, the inverse association between responsiveness to feedback and depressive symptoms was significant at higher levels (*β* = −0.16, *p* = 0.005) of perceived financial strain but not at lower and medium levels (*p*s > 0.106) (Figure 1b). The inverse association between context sensitivity and positive affect was only significant at higher levels (*β* = −0.13, *p* = 0.045) but not at lower and medium levels of perceived financial strain (*β* = 0.09, *p* = 0.242 and *β* = −0.20, *p* = 0.728, respectively) (Figure 2).

## 5. Discussion

In three studies, we developed and validated a vignette task for assessing regulatory flexibility of sustaining daily routines in the face of financial strain and tested its associations with psychological outcomes in separate samples of participants. The ability of sustaining daily routines was assessed in terms of context sensitivity and responsiveness to feedback detailed in the regulatory flexibility perspective [30]. Concurrent validity was demonstrated by the moderate correlations of context sensitivity and responsiveness to feedback of sustaining daily routines with the general ability of context sensitivity and social problem-solving ability. Discriminant validity was asserted by null and weak correlations of context sensitivity and responsiveness to feedback with measures assessing regular behavioral pattern and flexible regulation of emotion expression. Criterion-related validity was shown in the moderate inverse correlations of context sensitivity and responsiveness to feedback with PTSD symptoms. Consistent with our expectations, path analysis showed that context sensitivity was inversely associated with anxiety and depressive symptoms. Perceived financial strain moderated the inverse associations of context sensitivity and responsiveness to feedback with depressive symptoms and the inverse association of context sensitivity with positive effects.

### 5.1. Validity and Reliability of the Vignette Approach

This study is among the first to develop and use a novel method, i.e., a vignette task, to assess people’s coping with financial strain as a realistic and ecologically valid stressor. Previous studies on the association of subjective financial strain with mental health outcomes and coping processes demonstrate interrelationships more than causal relationships [6,7,8,9]. Psychological distress could increase subjective feeling of financial strain and negative perception of coping experiences. Vignette research is considered a hybrid of experimental and survey methods. Similar to experimental design, the independent variable can be operationalized into different conditions (i.e., vignettes) in the same manner as in experimental studies. Data collection relies on self-reported responses to questions about the vignettes comparable to a traditional survey design. Therefore, the current study has the strength of both high internal validity, referring to the degree to which changes in dependent variables (i.e., psychological distress and well-being) is attributable to changes in independent variables (i.e., sustainment of routines), and high external validity, referring to the high generalizability of the results to real-world situations [64]. The quasi-experimental vignette methodology that we used could be considered one feasible direction for enhancing internal validity of research on social determinants of mental health and the underlying social cognitive processes in response to financial difficulties [48].

Context sensitivity and responsiveness to feedback of sustaining daily routines were moderately positively associated with problem-solving ability, indicating the emphasis on cognitive processes that are needed for adopting effective strategies to regularize daily routines. The ability to sustain daily routines during financial strain was furthermore distinguished by null or weak correlations with measures assessing the regularity of daily routines and the flexible regulation of emotion expression. The moderate positive associations of context sensitivity and responsiveness to feedback in sustaining daily routines with general ability of context sensitivity demonstrated an overlap between the current vignette measures of task-specific regulatory flexibility and general scenario-based general measures of the same construct. Specifically, context sensitivity and responsiveness to feedback in daily routines sustainment was more strongly associated with higher sensitivity to the absence of contextual cues relative to sensitivity to the presence of them. Several kinds of dysfunction (e.g., depression and anxiety disorders) are characterized by insensitivity to cue absence [65,66]. Moreover, cue absence responses were found to be inversely associated with measures of psychopathology and stress [31]. Sensitivity to cue presence could be more related to perception of contextual demands, whereas sensitivity to cue absence could be related to more clarity of genuine contextual demands, inattention to false demands, and thus less psychological distress. 

### 5.2. Sustainment of Daily Routines in Adaptation to Financial Strain

Our findings extend the current literature on context sensitivity and responsiveness to feedback to studying daily functioning during financial difficulties. Most if not all of the previous studies have investigated context sensitivity and responsiveness to feedback with regard to emotion regulation. Emotion context insensitivity has been found to be associated with several emotion disorders, most notably anxiety and depression [66,67]. Previous studies have also reported supportive evidence on the adaptive role of emotion context sensitivity in adjustment to severe stressful life events including loss of significant others [67], chronic illness [68], and emotional disorders [66,69]. We investigated the relationships between context sensitivity and responsiveness to feedback in sustaining daily routines with psychological distress and wellbeing under financial difficulties, which may be more relatable to people adapting to chronic stressful conditions. 

A significant inverse association was found between context sensitivity and positive affect at higher levels of perceived financial strain while such associations were non-significant at lower and medium levels of perceived financial strain. People perceiving higher levels of financial strain are more likely to be have a lower socioeconomic status and fewer coping resources [70]. Even though they are sensitive enough to detect situational demands, scarcity of coping resources could have limited them from corresponding behaviors for sustaining daily routines, which, in turn, result in unsuccessful regulatory attempts and diminished psychological well-being [71,72,73]. The inverse associations of context sensitivity and responsiveness to feedback with depressive symptoms were stronger at higher and medium levels relative to lower levels of perceived financial strain, consistent with previous evidence in emotion regulation [74], coping strategies [75,76], and states of mind such as cognitive control [77]. Although context sensitivity was inversely associated with positive affect at higher levels of perceived financial strain, in general, context sensitivity and responsiveness to feedback were linked with lower anxiety and depressive symptoms. Cultivating regular daily routines in everyday life may be a simple and effective intervention in reducing psychological distress [78,79,80,81], especially for persons with less socioeconomic resources (e.g., low savings, no home ownership) in times with multiple large-scale stressors such as civil unrest and pandemic [82].

### 5.3. Limitations

Several limitations of this study should be noted. First, our findings were generated from four sets of MTurk crowdsourcing data (*n* = 1685). There is supportive evidence on the representativeness, reliability, and validity of data collected on MTurk [49,50,51]. We also restricted one participation to each MTurk Worker ID in order to minimize repeated participations of the same person. What we could not control for is that people could have more than one MTurk Worker ID and repeated participations could inadvertently influence the validity of the collected data. Nevertheless, the three studies addressed cohesive but relatively distinct topics and thus could be minimally impacted by multiple participations. Second, this study started off with investigating the ability to sustain daily routines amid financial strain, but the participants consisted of people with varying socioeconomic statuses. More investigation is needed to examine whether the current findings are generalizable and even more applicable to people with objective financial strain [2,3], as we found significant moderating effects of subjective financial strain in the sustainment-health link. Third, scenarios (i.e., jobs, lifestyle) and daily routines in the vignette task were formulated based on the demographics of the USA, and all data were collected in the USA. An important point to note is that the associations among financial strain, daily routines, and mental health could be different between more and less developed parts of the world, and therefore the current findings might not hold true in non-Western societies. For example, within the collectivistic sociocultural context in Nigeria and Ghana, people show different reactions to family routines disruptions compared with people within more individualistic sociocultural contexts in Western societies [83], suggesting differential mechanisms of regularizing daily routines for mental health. These could limit the transferability of the findings to populations living in other sociocultural contexts. 

## 6. Conclusions and Implications

Notwithstanding the above limitations, our findings on regulating daily routines for mental health in financial difficulties provide a feasible cost-effective approach for developing scalable behavioral interventions that could be conducted by trained non-specialists, digital treatments, and guide self-help for the early management of potential mental health disorders [84]. First, validated instruments can be used for assessing specific dimensions of daily routines that have been disrupted by continuous financial difficulties and common mental health outcomes including anxiety and depressive symptoms. Then, based on the data, a vignette task can be adopted as an objective measurement of individuals’ ability to manage those affected routines. Individuals at risk of poorer psychological adjustment could be instructed on the dimensions of the daily routines that they should attend to and regulate and how they could possibly manage their routines sequentially for better mental health.

## Figures and Tables

**Table 1:** Demographic characteristics of the four samples.

Demographic Variables	Sample			
	Sample 1	Sample 2	Sample 3	Sample 4
	*n* = 356	*n* = 282	*n* = 509	*n* = 538
**Mean age (SD)**	34.5 (10.9)	34.0 (10.7)	33.2 (9.7)	35.2 (10.9)
**Gender**				
Male	190 (53.4%)	184 (65.2%)	318 (62.5%)	278 (51.6%)
Female	166 (46.6%)	95 (33.7%)	187 (36.7%)	258 (48.0%)
Other	0 (0.0%)	3 (1.1%)	4 (0.8%)	2 (0.4%)
**Annual income (US $)**				
0–19,999	98 (27.5%)	50 (17.7%)	130 (25.5%)	128 (23.8%)
20,000–39,999	91 (25.6%)	94 (33.3%)	156 (30.6%)	162 (30.1%)
40,000–59,999	84 (23.6%)	64 (22.7%)	110 (21.6%)	113 (21.0%)
60,000–79,999	46 (12.9%)	36 (12.8%)	68 (13.4%)	81 (15.1%)
80,000–99,999	16 (4.5%)	22 (7.8%)	27 (5.3%)	29 (5.4%)
100,000+	21 (5.9%)	16 (5.7%)	18 (3.5%)	25 (4.6%)
**Marital status**				
Single	134 (37.6%)	124 (44.0%)	281 (55.2%)	211 (39.2%)
Married	195 (54.8%)	146 (51.8%)	198 (38.9%)	287 (53.3%)
Divorced	23 (6.5%)	12 (4.3%)	27 (5.3%)	38 (7.1%)
Widowed	4 (1.1%)	0 (0.0%)	3 (0.6%)	2 (0.4%)
**Employment status**				
Full-time	256 (71.9%)	232 (82.3%)	373 (73.3%)	382 (71.0%)
Part-time	57 (16.0%)	25 (8.9%)	80 (15.7%)	86 (16.0%)
Unemployed	16 (4.5%)	12 (4.3%)	38 (7.5%)	29 (5.4%)
Housewife	17 (4.8%)	7 (2.5%)	13 (2.6%)	33 (6.1%)
Retired	10 (2.8%)	6 (2.1%)	5 (1.0%)	8 (1.5%)
**Educational attainment**				
High school diploma or equivalent	32 (9.0%)	32 (11.3%)	62 (12.2%)	59 (11.0%)
Some college	82 (23.0%)	57 (20.2%)	135 (26.5%)	149 (27.7%)
College diploma	102 (28.7%)	67 (23.8%)	188 (36.9%)	191 (35.5%)
Some graduate school	26 (7.3%)	16 (5.7%)	31 (6.1%)	22 (4.1%)
Graduate degree	114 (32.0%)	110 (39.0%)	93 (18.3%)	117 (21.7%)
**Race (Non-mutually exclusive)**				
Hispanic	69 (19.4%)	50 (17.7%)	95 (18.7%)	82 (15.2%)
Asian	115 (32.3%)	94 (33.3%)	108 (21.2%)	111 (20.6%)
White	204 (57.3%)	168 (59.6%)	334 (65.6%)	378 (70.3%)
African American	20 (5.6%)	13 (4.6%)	63 (12.4%)	45 (8.4%)
American Indian	24 (6.7%)	9 (3.2%)	32 (6.3%)	22 (4.1%)
Hawaiian/other Pacific Islander	2 (0.6%)	0 (0.0%)	3 (0.6%)	2 (0.4%)

**Table 2:** Pearson correlations between regulatory flexibility in daily routines and self-reported instruments in Study 2.

	Sample Size	Context Sensitivity	Responsiveness to Feedback
**Concurrent validity**			
Sensitivity to the absence of contextual cues	282	0.497 ***	0.357 ***
Sensitivity to the presence of contextual cues	282	0.389 ***	0.341 ***
Social problem-solving ability	282	0.497 ***	0.403 ***
**Discriminant validity**			
Regularity of daily routines	282	0.127 *	0.152 *
Flexible regulation of emotion expression	282	0.050	0.008
**Criterion-related validity**			
PTSD symptoms	509	−0.452 ***	−0.389 ***

* *p* < 0.05, *** *p* < 0.001.

**Table 3:** The characteristics of the perceived financial strain of the sample (*n* = 538).

**How Often Do You Not Have Enough Money to Afford the Following?**	**Almost Never/Never (%)**	**Sometimes (%)**	**Often (%)**	**Almost Always (%)**
Food	39.2	15.4	17.9	27.5
Medical care	30.9	23.2	19.5	26.4
Clothing	30.9	27.3	18.2	23.6
Family leisure activities	23.2	33.3	22.9	20.6
**How do your finances usually work out at the end of the month?**	**Some money left over (%)**	**Just enough money to make ends meet (%)**	**Not enough money to make ends meet (%)**	
	34.9	52.6	12.5	

**Table 4:** Descriptive statistics and correlations of the study variables in Study 3.

Variable	1. Context Sensitivity	2. Responsiveness to Feedback	3. Perceived Financial Strain	4. Anxiety Symptoms	5. Depressive Symptoms	6. Positive Affect	7. Life Satisfaction
**1**	–						
**2**	0.608 ***	–					
**3**	−0.240 ***	−0.108 *	–				
**4**	−0.346 ***	−0.247 ***	0.164 ***	–			
**5**	−0.532 ***	−0.382 ***	0.340 ***	0.608 ***	–		
**6**	−0.078	−0.035	0.093 *	−0.277 ***	−0.067	–	
**7**	−0.102 *	−0.105 *	0.026	−0.297 ***	−0.125 **	0.598 ***	–
**Range**	−0.52–0.98	−0.58–0.92	−1.19–1.50	6–22	0–27	10–50	5–20
**Mean (*SD*)**	0.53 (0.30)	0.42 (0.29)	0 (0.72)	10.67 (3.74)	6.95 (7.06)	32.94 (8.91)	13.72 (3.63)
**Skewness**	−0.977	−0.920	−0.145	0.612	0.865	−0.032	−0.443
**Kurtosis**	0.542	0.571	−1.094	−0.228	−0.265	−0.524	−0.143

* *p* < 0.05, ** *p* < 0.01, *** *p* < 0.001.

## Data Availability

The data presented in this study are available on request from the corresponding author.

## Supplementary Materials

The following are available online at https://www.mdpi.com/1660-4601/18/6/3103/s1. Table S1. Summary of the three studies. Table S2. Associations between regulatory flexibility in daily routines and self-reported instruments in Study 2 with gender being controlled for.

## Appendix A

**Table A1:** A sample of complete vignette.

Julie is a 57-year-old woman who works part-time as a seamstress. She just moved across the country to be closer to her daughter since her husband died last year. Julie is struggling to find regular work and cannot keep up with her rent and other bills. She used to shower regularly. Now, she has only been showering on occasion. She used to cook regularly. Now, she never finds time for a proper meal.
**Initial ranking**. If you were this person, realistically, how would you respond to this situation? Please rank the following items from 1 to 5 in order of importance with 1 being the most important and 5 being the least important: I would work on showering more regularly. I would start cooking meals more again. I would work on getting more regular exercise. I would start spending more time on relaxation. I would not do anything.
**Sub-question 1**. If after doing those things you find that you **feel better physically and mentally**, how would you prioritize them now? Please rank the following from 1 to 5 in order of importance with 1 being the most important and 5 being the least important: I would work on showering more regularly. I would start cooking meals more again. I would work on getting more regular exercise. I would start spending more time on relaxation. I would not do anything.
**Sub-question 2**. If after doing those things you find that you **do not feel better physically and mentally**, how would you prioritize them now? Please rank the following from 1 to 5 in order of importance with 1 being the most important and 5 being the least important: I would work on showering more regularly. I would start cooking meals more again. I would work on getting more regular exercise. I would start spending more time on relaxation. I would not do anything.
