# Supplementary material for: Regulatory Flexibility of Sustaining Daily Routines and Mental Health in Adaptation to Financial Strain: A Vignette Approach
Source: Int J Environ Res Public Health. 2021 Mar 17;18(6):3103. doi: 10.3390/ijerph18063103 (PMC8002825; doi:10.3390/ijerph18063103)
Supplement: Supplementary file 1 [file ijerph-18-03103-s001.pdf]

International Journal of
Environmental Research and Public Health

MDPI

Supplementary Materials

Table S1. Summary of the three studies.

	Study 1	Study 2	Study 3
**Aims**	To develop the vignette task for subsequent studies.	To examine the validity of regulatory flexibility of sustaining daily routines.	To investigate the association between sustainment of routines and mental health and the moderating effect of subjective financial strain on the association.
**Description of the Study**	Vignettes were initially drafted, tested, and modified with reference to the pilot data on the relevance of the scenarios and response options among a community sample.	The correlations between regulatory flexibility of sustaining daily routines, calculated in terms of context sensitivity and responsiveness to feedback, and self-reported instruments were examined.	The associations of individual differences in the ability of context sensitivity and responsiveness to feedback of sustaining daily routines for financial strain with psychological distress and well-being, and the moderating effects of subjective financial strain on the associations, were tested simultaneously by path analysis.
**Findings**	A total of 48 statements were selected for the vignette task.	Concurrent validity was demonstrated by the moderate correlations of regulatory flexibility of sustaining daily routines with the general ability of context sensitivity and social problem-solving ability. Discriminant validity was asserted by null and weak correlations of context sensitivity and responsiveness to feedback with measures assessing regular behavioral pattern and flexible regulation of emotion expression. Criterion-related validity was shown in the moderate inverse correlations of context sensitivity and responsiveness to feedback with PTSD symptoms.	Context sensitivity was inversely associated with anxiety and depressive symptoms. Perceived financial strain moderated the inverse associations of context sensitivity and responsiveness to feedback with depressive symptoms and the inverse association of context sensitivity with positive affect.

Table S2. Associations between regulatory flexibility in daily routines and self-reported instruments in Study 2 with gender being controlled for.

	**Context Sensitivity**	**Responsiveness to Feedback**
**Concurrent validity**		
Sensitivity to the absence of contextual cues	0.503***	0.358***
Sensitivity to the presence of contextual cues	0.390***	0.335***
Social problem-solving ability	0.498***	0.403***
**Discriminant validity**		
Regularity of daily routines	0.146*	0.165**
Flexible regulation of emotion expression	0.043	0.001
**Criterion-related validity**		
PTSD symptoms	−0.438***	−0.347***

* $p < 0.05$, ** $p < 0.01$, *** $p < 0.001$. Standardized coefficients were reported.
